# Hepatitis C treatment strategies in prisons: A cost-effectiveness analysis

**DOI:** 10.1371/journal.pone.0245896

**Published:** 2021-02-11

**Authors:** Jisoo A. Kwon, Georgina M. Chambers, Fabio Luciani, Lei Zhang, Shamin Kinathil, Dennis Kim, Hla-Hla Thein, Willings Botha, Sandra Thompson, Andrew Lloyd, Lorraine Yap, Richard T. Gray, Tony Butler

**Affiliations:** 1 The Kirby Institute, UNSW Sydney, Sydney, NSW, Australia; 2 National Perinatal Epidemiology and Statistics Unit (NPESU), Centre for Big Data Research in Health and School of Women’s and Children’s Health, UNSW Sydney, Sydney, NSW, Australia; 3 School of Medical Sciences, Faculty of Medicine, UNSW, Sydney, NSW, Australia; 4 The Melbourne Sexual Health Centre, Alfred Health, Carlton, Melbourne, VIC, Australia; 5 Toronto Health Economics and Technology Assessment Collaborative (THETA), Toronto General Hospital Research Institute, University of Toronto and University Health Network, Toronto, ON, Canada; 6 RTI Health Solutions, Research Triangle Park, NC, United States of America; 7 Combined Universities of Rural Health, Geraldton, WA, Australia; Centers for Disease Control and Prevention, UNITED STATES

## Abstract

In Australian prisons approximately 20% of inmates are chronically infected with hepatitis C virus (HCV), providing an important population for targeted treatment and prevention. A dynamic mathematical model of HCV transmission was used to assess the impact of increasing direct-acting antiviral (DAA) treatment uptake on HCV incidence and prevalence in the prisons in New South Wales, Australia, and to assess the cost-effectiveness of alternate treatment strategies. We developed four separate models reflecting different average prison lengths of stay (LOS) of 2, 6, 24, and 36 months. Each model considered four DAA treatment coverage scenarios of 10% (status-quo), 25%, 50%, and 90% over 2016–2045. For each model and scenario, we estimated the lifetime burden of disease, costs and changes in quality-adjusted life years (QALYs) in prison and in the community during 2016–2075. Costs and QALYs were discounted 3.5% annually and adjusted to 2015 Australian dollars. Compared to treating 10% of infected prisoners, increasing DAA coverage to 25%, 50%, and 90% reduced HCV incidence in prisons by 9–33% (2-months LOS), 26–65% (6-months LOS), 37–70% (24-months LOS), and 35–65% (36-months LOS). DAA treatment was highly cost-effective among all LOS models at conservative willingness-to-pay thresholds. DAA therapy became increasingly cost-effective with increasing coverage. Compared to 10% treatment coverage, the incremental cost per QALY ranged from $497-$569 (2-months LOS), -$280–$323 (6-months LOS), -$432–$426 (24-months LOS), and -$245–$477 (36-months LOS). Treating more than 25% of HCV-infected prisoners with DAA therapy is highly cost-effective. This study shows that treating HCV-infected prisoners is highly cost-effective and should be a government priority for the global HCV elimination effort.

## Introduction

Nearly 200,000 Australians have hepatitis C infection, a leading cause of liver-related deaths, with around 400 deaths per 100,000 population are attributed to hepatitis C virus (HCV) infection in 2015 [1, 2]. Approximately two-thirds of new infections fail to resolve and persist for life, resulting in an estimated 6,000 new cases of chronic HCV infection each year [2]. HCV is the leading indication for liver transplantation and liver-related deaths in Australia and without treatment, approximately 20–30% of those with chronic HCV will progress to cirrhosis, liver failure or hepatocellular carcinoma [3]. The risk of acquiring infectious disease in the prison setting is higher than in the general population including high rates of exposure to blood-borne viral infections such as HCV.

Recent advances in HCV treatment have revolutionised the clinical management of viral hepatitis. The development of direct-acting antiviral (DAA) therapy for HCV provides a safe and highly effective cure rate of over 95% [4–8]. In March 2016, the Australian government provided highly subsidized access to DAA therapy for people living with HCV, without restrictions on liver disease stage, drug and alcohol use, or prescriber type, with specific inclusion of access for prisoners [9].

Prisoner populations are characterised by high rates of exposure to blood-borne viral infections such as HCV [10–15]. The prevalence of HCV antibodies in Australian prisons is high, with one in five inmates chronically infected [16]. The most recent prevalence estimates suggest that between 7,500 and 10,000 prisoners in Australia, are HCV antibody positive and approximately 7,500 chronically infected with HCV [17]. A recent meta-analysis of HCV incidence studies among prisoners revealed a mean incidence of 11.4 cases per 100 person-years in the New South Wales, Australia [15]. The prisoner population in Australia, as in other countries, comprised mostly men with less than one in 10 prisoners in Australia identifying as female [18]. Some gender differences in the prevalence of HCV are apparent [19].

Transmission of HCV in prison is strongly associated with injecting drug use and sharing contaminated injecting equipment [14]. Around 8 in 10 people who inject drugs (PWIDs) spend time in prison at some time in their lives with many cycling through on multiple occasions [20]. Prisons can be regarded as an enclosed network of facilities within which prisoners are frequently moved between sites due to changes in security classifications, to attend court approaches, for safety reasons, or to obtain medical treatment. Among PWID in New South Wales (NSW) prisons, Australia, the length of stay (LOS) before being released, is typically short (average 7–9 months). Although prison-based hepatitis services have been established in NSW for many years, the short incarceration period rendered treatment of prisoners with interferon-based therapies difficult due to the 6 to 12-month treatment period, in addition to the challenge for both the patients and service providers in managing the substantial side-effects of these older treatments [21, 22]. However, the introduction DAA therapies, which typically only require an 8 to 12-week course of once-daily, oral, very low side-effect and highly curative treatment with a 95% cure rate [4–6] has provided an opportunity to cure those with HCV infection whilst in prison, and so reduce the overall population burden of HCV infection in the community. As such, prisons provide a potentially important setting to reduce the overall burden of HCV infection both in terms of treatment and prevention [23].

We developed a mathematical model that describes HCV transmission dynamics in the NSW prison setting, accounting for key risk behaviours (e.g. injecting drug use), prison dynamics (e.g. duration of incarceration), natural history of HCV-related liver disease, and healthcare costs associated with DAA treatment interventions and burden of living with HCV. Mathematical models are useful tools for understanding complex dynamical systems, such as infectious disease epidemics and are typically used to study the mechanisms of disease spread and to predict future disease outbreaks. Future transmission of infectious disease epidemics such as HCV are difficult to predict, but by using mathematical models to identify dominant factors of disease transmission, future patterns of disease spread and impact can be predicted. Mathematical models provide transparency, and the calibration phase of modeling enables epidemiological assumptions to be tested by comparing model results with observed patterns [24, 25]. By conducting a cost-effectiveness analysis using the output of mathematical models, in this case implementation of DAA treatment regimes in the prison setting, the costs and effects of interventions on targeting populations and of changing the level of intervention coverage can also be evaluated.

## Materials and methods

The mathematical model is compartmentalized and implemented as a system of ordinary differential equations detailing sub-populations of prison inmates according to injecting drug use, sharing of injecting apparatus, and liver disease status. The DAA treatment interventions were modelled for 30 years in prison (2016–2045), with healthcare costs and quality-adjusted life years (QALYs) tracked for a further 30 years post prison (2046–2075) to capture long-term costs and health gains from high cure rates. We assumed status quo for 2016 with HCV treatment coverage providing DAA treatment to 10% the prison population regardless of liver disease stage (F0 to F4) and genotype, using a nurse-led model of care [26]. The model parameters are described in S1 and S2 Tables in S1 File.

**Fig 1:**
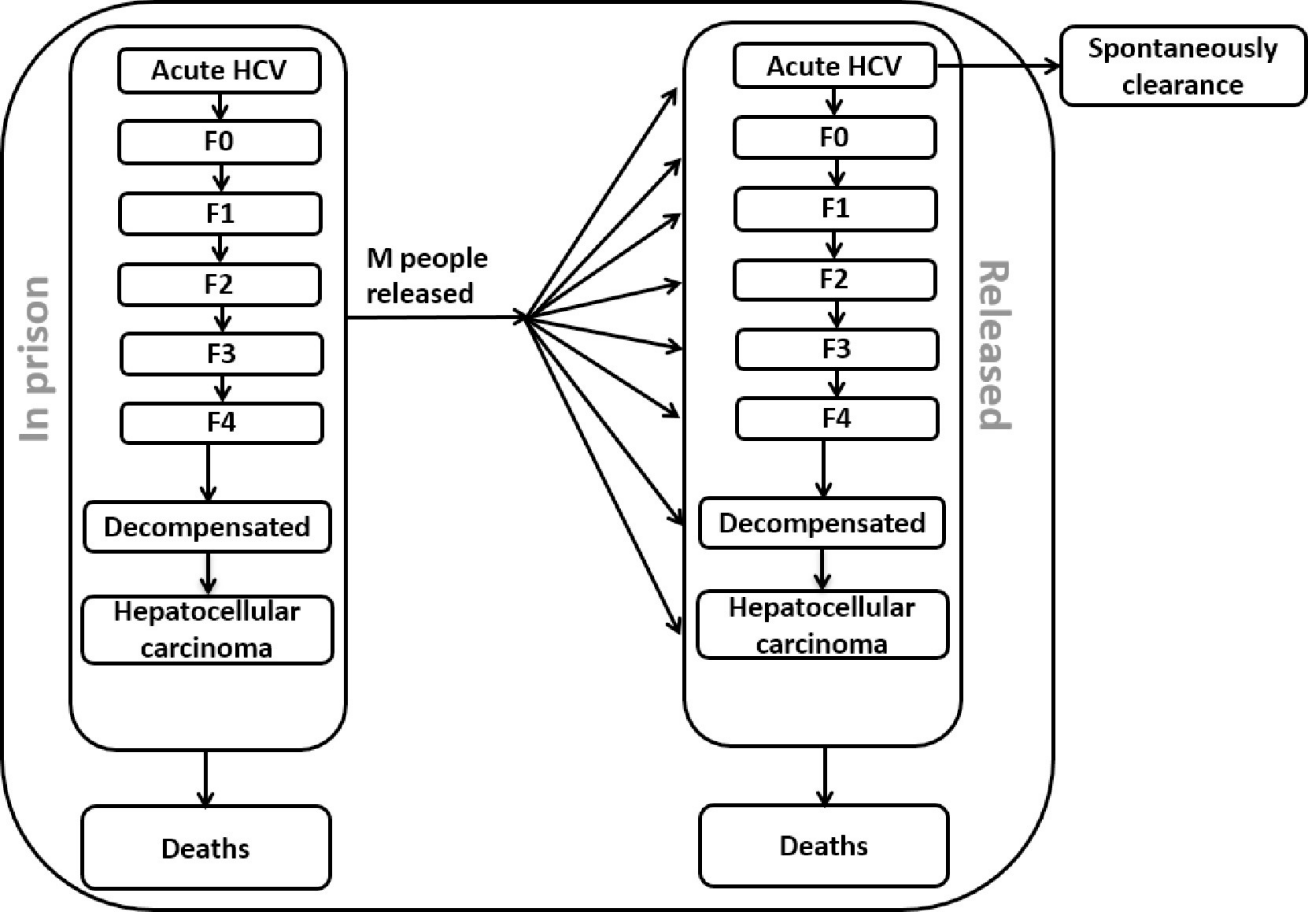
Schematic diagram of HCV disease progression among prisoners in Australia. Each arrow represents the rate of change in the number of people in the population moving from one health state compartment to another health state compartment.

The model tracks the acute stage (A) of HCV infection which results in spontaneous clearance in 25% [27] of cases, each fibrosis stage (F0, F1, F2, F3 and F4 (compensated cirrhosis)), decompensated cirrhosis and hepatocellular carcinoma, and the number of people who received liver transplantation due to chronic HCV (See schematic diagram, Fig 1). The population of people who leave prison after incarceration is tracked up to 2075 to account for the lifetime costs and changes in health-related quality of life of those treated and not treated. All simulations and analyses were performed in MATLAB 2016a (The Mathworks, Natick, Massachusetts, USA) and Microsoft Excel (Redmond, WA).

### Model calibration

The model calibration was conducted using available data on HCV incidence and prevalence (S1 Table in S1 File). The calibration was based on an observed decline in HCV antibody prevalence between 2001 and 2009 for the total prisoner population, but with a sustained HCV-Ab prevalence of 70%+ among PWID [19].

### Treatment and length of stay scenarios

Prisoners in NSW move frequently between prison locations, and between the community and prisons. The analysis was performed on 4 settings with an average LOS of 2, 6, 24, 36 months respectively. The treatment scenarios were then applied for each LOS model from 2016 to 2045, with 10% (status-quo), 25%, 50%, and 90% treatment coverage [21].

### Costs of treatment and care

The costs of treatment including the cost of the DAA medication and delivery under a nurse-led model of care were assigned according to the perspective of the NSW Justice Health and Forensic Mental Health Network and Corrective Services NSW, the former providing medical services to prisoners in NSW and the latter security. Salaries for nursing and medical staff to administer the treatment program and pharmacists’ were sourced from Australian Government’spublished pay scales [28]. The cost of the DAAs has been estimated at $15,000 Australian dollars (AUD) per patient for a 12-week course (consultation with an expert) [29], and the cost of administration under the nurse-led model of care as $666 AUD based in bottom up costing methods incorporating staff time and consumables.

Accordingly post-treatment, the annual public healthcare resources consumed for lifetime care (burden of disease) associated with HCV infection and sequalae were calculated from a healthcare perspective. Micro-costing methods were used to determine the unit costs and volumes of the annual care of patients with HCV stages F0-F3 (first year and subsequent years), F4 (compensated cirrhosis), decompensated cirrhosis, liver failure, and hepatocellular carcinoma. Costs included specialist visits, clinical care nurse visits, pathology, medical procedures, and hospital admissions. Unit costs were obtained from the Australian Medical Benefits Scheme(assuming 100% government benefits) for medical services, procedures, and pathology tests. Medication costs were sourced from the Australian Pharmaceutical Benefits Scheme [30, 31]. Inpatient medical procedures and hospital stays were sourced from the National Hospital Cost Data Collection using Australian Refined Diagnosis Related Groups (AR-DRG 5.1) [32]. Costs were adjusted to 2015 AUD and discounted at 3.5% annually to reflect the future long-term value of the costs incurred in treating and living with HCV infection [33].

### Cost-effectiveness analysis

The cost-effectiveness analysis applied the costs and QALYs to the forecasted epidemiological outcomes using the trajectory of HCV infection among Australian prison detainees over a treatment period of 2016–2045, and follow-up period to 2075. Health state utilities used in the derivation of the QALYS were obtained from the literature and attached to each liver disease stage [34]. QALYs were also discounted by 3.5% per annum. The costs and utility weights used in the analysis can be found in S2 Table in S1 File. The key epidemiological indicators for the cost-effectiveness analysis included the number of new HCV cases, HCV-related deaths and number of people on treatment.

Incremental cost-effectiveness ratios (ICERs) were reported separately for the four LOS models (2, 6, 24, 36 months). Within each model a pair-wise comparison was conducted between 10% DAA coverage with 25%, 50% and 90% coverage of HCV-infected inmates, resulting in 12 scenarios. The ICER was expressed as the incremental change in total costs and QALYs relative to 10% DAA coverage for each LOS scenario. Strategies were considered to be cost-effective if they generated an ICER less than the willingness-to-pay threshold (lambda) considered to represent good value for money for healthcare interventions in Australia ($28,000 per QALY gained) [35].

The uncertainty around the costs and QALYs was explored through probabilistic sensitivity analysis (PSA) [36]. This was undertaken using 5,000 Monte Carlo simulations which repeatedly creates random data through bootstrapping. A Gamma probability distribution was assigned to the cost parameter computed from the variation of costs in treatment coverage (10%, 25%, 50%, 90%) [37]. The QALYs were assigned a normal distribution with standard deviations calculated from QALYs resulting from each treatment coverage. The PSA allowed the estimation of 95% CI for the ICERs from the bootstrapped replications of data using the percentile approach with a lower and upper percentile of 0.025 and 0.975, respectively [37]. Bootstrapping was performed in Microsoft Excel version 16 and implemented using a Microsoft Excel macro.

A Net Monetary Benefit (NMB) approach was also used allowing multiple alternatives to be assessed for cost-effectiveness without requiring repeated pair-wise comparisons and allows the most cost-effective alternative to be easily identified for different willingness-to-pay thresholds. The NMB is defined as: (lambda x (incremental effect)–(incremental cost)). NMB is calculated by assuming a threshold for willingness-to-pay, then converting QALYs into the corresponding dollar values. The cost associated with each treatment strategy is then subtracted by the baseline scenarios, resulting in the net benefit of each strategy expressed in the monetary units.

## Results

**Fig 2:**
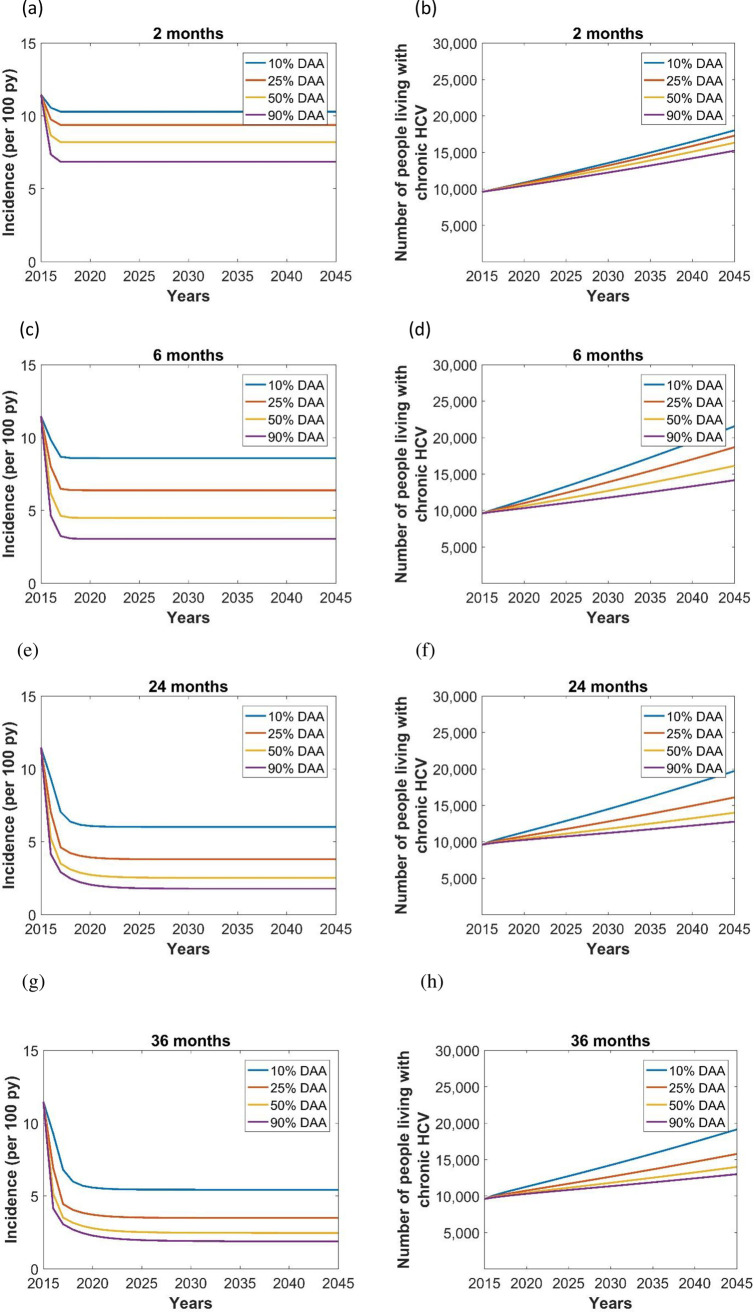
Effect of length of stay (LOS) in prison (2 months LOS (a,b), 6 months LOS (c,d), 24 months LOS (e,f), 36 months LOS (g,h)) and treatment coverage (blue lines: 10% DAA, red lines: 25% DAA, orange lines: 50% DAA, purple lines: 90% DAA) on the incidence (per 100 py) and prevalence of chronic HCV in prison.

The model predicted a sharp decline in new HCV infections in all LOS scenarios (Fig 2). Simulations with an increased treatment coverage reduced HCV incidence rate by 9–33% (2-months LOS), 26–65% (6-months LOS), 37–70% (24-months LOS), and 35–65% (36-months LOS), showing that HCV treatment coverage was the major factor in reducing HCV infections particularly in the longer LOS models of 6 or greater months.

Analysis of prevalence showed a linear increase over time, regardless of the average LOS, and lower prevalence with higher treatment coverage (Fig 2). The incidence and prevalence of chronic HCV remained relatively high and showed less impact of treatment coverage in the 2 months LOS model because of the high turn-over of HCV infected patients in these prisons. Similarly, the prevalence of chronic HCV was lowest with 36 months LOS, because the lower number of people coming into the population corresponds to a reduced incidence rate (Table 1). Except for the 2 months LOS, from 50% DAA coverage, increasing LOS did not play a major role in reducing the prevalence of HCV, but increases in treatment coverage reduced the prevalence of HCV. Chronic HCV prevalence was 18,045, 21,573, 19,731, and 19,162 with 10% DAA coverage in 2 months, 6 months, 24 months, and 36 months LOS scenarios, respectively. We estimated that an increase in DAA treatment coverage from 10% to 90% would avert 2,793 (15% reduction), 7,398 (34% reduction), 6,943 (35% reduction), and 6,133 (32% reduction) HCV cases during 2015–2045 in the 2 months, 6 months, 24 months, and 36 months LOS, respectively (Table 1).

**Table 1:** Estimated HCV-related outcomes with treatment scenarios and length of stay (best estimate).

**Incidence rate (2015, per 100 py)**	**11**			
**People living with chronic HCV (2015)**	**9,623**			
	**10% DAA**	**25% DAA**	**50% DAA**	**90% DAA**
**Incidence rate (2045, per 100 py)**				
2 months length of stay	10.3	9.4	8.2	6.9
6 months length of stay	8.6	6.4	4.5	3.0
24 months length of stay	6.0	3.8	2.5	1.8
36 months length of stay	5.4	3.5	2.5	1.9
**People living with chronic HCV (2045)**				
2 months length of stay	18,045	17,311	16,351	15,252
6 months length of stay	21,573	18,700	16,149	14,175
24 months length of stay	19,731	16,117	14,017	12,788
36 months length of stay	19,162	15,806	14,023	13,029
**Cumulative liver-related deaths (2015–2045)**				
2 months length of stay	27,104	27,075	27,042	27,008
6 months length of stay	32,344	32,110	31,936	31,817
24 months length of stay	33,140	32,476	32,151	31,981
36 months length of stay	32,679	31,917	31,573	31,400

PY, person-years; HCV, hepatitis C virus; DAA, direct-acting antiviral.

The number of NSW male prisoners treated between 2016–2045 ranged from 21,473 in the 36-month average LOS in prison with 10% coverage to 176,985 prisoners in the 2-month average LOS in prison with 90% coverage. The total cost attributed to the treatment of HCV-infected prisoners over a 30-year treatment period (2016–2045) plus the lifetime cost of care for a further 30 years ranged from $3,469 to $3,997 million with 10% DAA coverage (Table 2). Increasing coverage to 90% of infected prisoners during the same period, would incur an additional $515-$1,061 million.

**Table 2:** Cost-effectiveness analysis of HCV treatment in prison, by length of stay and DAA treatment coverage, AUD 2015, mean (lower-upper).

Scenarios	Cumulative number treated (2016–2045)	Total Cost	Total QALYs	ICER	NMB ($M)	NMB ($M)
		($M, discounted, 2016–2075)	($M, discounted, 2016–2075)		WTP = $28,000	WTP = $100,000
**2 months length of stay**						
**10% DAA**	22,388	$3,680 $(3,637–3,723)	1.36 (1.35–1.38)	-	-	-
**25% DAA**	53,972	$3,861 $(3,823–3,898)	1.73 (1.71–1.75)	$497 (517–473)	$10,031 (9,857–10,226)	$36,290 (35,682–36,974)
**50% DAA**	103,206	$4,187 $(4,137–4,234)	2.32 (2.29–2.35)	$532 (534–526)	$26,195 (25,749–26,710)	$94,859 (93,247–96,709)
**90% DAA**	176,985	$4,741 $(4,684–4,798)	3.23 (3.19–3.28)	$569 (572–566)	$51,212 (50,252–52,164)	$185,631 (182,164–189,064)
**6 months length of stay**						
**10% DAA**	24,339	$3,997 $(3,950–4,044)	1.52 (1.51–1.53)	-	-	-
**25% DAA**	54,399	$3,919 $(3,882–3,958)	1.80 (1.78–1.81)	-$280 (-308–251)	$7,817 (7,668–7,961)	$27,718 (27,211–28,209)
**50% DAA**	99,057	$4,065 $(4,020–4,112)	2.29 (2.26–2.32)	$88 (87–92)	$21,487 (21,059–21,881)	$76,914 (75,389–78,323)
**90% DAA**	166,251	$4,512 $(4,458–4,562)	3.11 (3.07–3.16)	$323 (318–325)	$44,133 (43,286–45,013)	$158,940 (155,899–162,092)
**24 months length of stay**						
**10% DAA**	22,054	$3,559 $(3,518–3,599)	1.41 (1.40–1.42)	-	-	-
**25% DAA**	48,538	$3,462 $(3,230–3,492)	1.64 (1.62–1.65)	-$432 (-463–396)	$6,386 (6,289–6,563)	$22,557 (22,236–23,164)
**50% DAA**	90,429	$3,686 $(3,644–3,727)	2.13 (2.10–2.15)	$178 (175–180)	$19,868 (19,494–20,294)	$71,284 (69,946–72,807)
**90% DAA**	156,342	$4,222 $(4,172–4,274)	2.97 (2.93–3.01)	$426 (425–428)	$42,945 (42,126–43,815)	$155,083 (152,134–158,218)
**36 months length of stay**						
**10% DAA**	21,473	$3,469 $(3,432–3,506)	1.41 (1.40–1.43)	-	-	-
**25% DAA**	47,743	$3,407 $(3,377–3,437)	1.64 (1.63–1.66)	-$245 (-274–297)	$6,455 (6,334–6,589)	$22,893 (22,480–23,353)
**50% DAA**	89,920	$3,663 $(3,624–3,705)	2.14 (2.12–2.17)	$268 (267–269)	$20,197 (19,795–20,589)	$72,631 (71,190–74,042)
**90% DAA**	156,660	$4,226 $(4,175–4,276)	3.00 (2.96–3.04)	$477 (477–476)	$43,367 (42,822–44,495)	$157,790 (154,847–160,891)

**Note:** ICER = Incremental costs/incremental QALYs, NMB = Willingness to pay * incremental QALYs–incremental costs.

HCV, hepatitis C virus; DAA, direct-acting antiviral; QALYs, quality-adjusted life years; ICER, incremental cost-effectiveness ratio; NMB, Net Monetary Benefit; WTP, willingness-to-pay; M, million.

**Fig 3:**
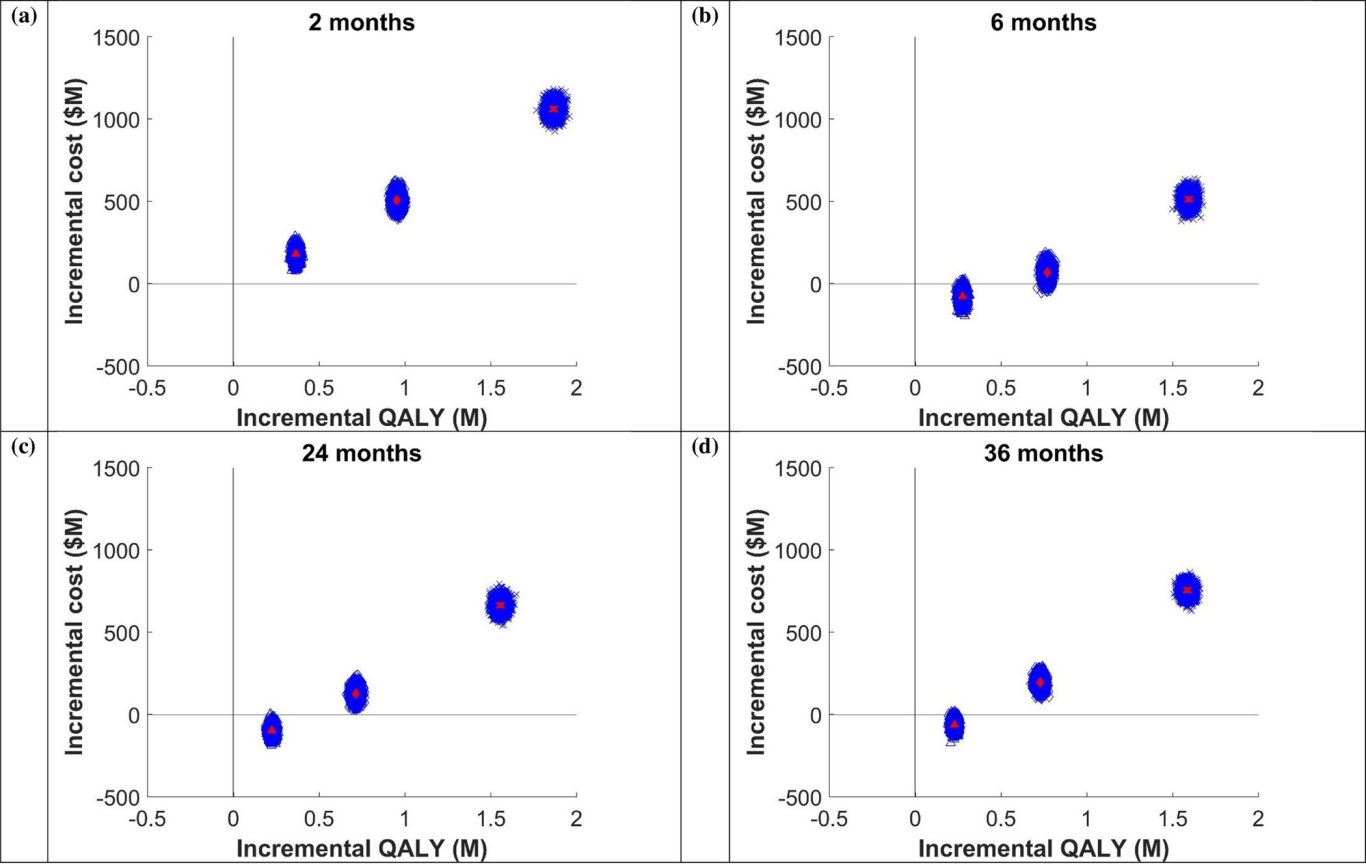
ICER plane for (a) 2 months, (b) 6 months, (c) 24 months, and (d) 36 months length of stay. Triangle shape represents 25% of people receive DAA, diamond shape represents 50% DAA, and cross shape represents 90% DAA.

Treating 25% of infected prisoners was less costly and more effective for all LOS except 2 months LOS. The cost per QALY gained increased with increasing DAA coverage. For example, for the 6-month average LOS prison model; compared to 10% treatment coverage of HCV infected prisoners, treating 25%, 50%, and 90% of infected prisoners resulted in ICERs of -$280, $88, and $323, respectively. Similarly, for the 36-month average LOS prison model, treating 25%, 50%, and 90% of infected prisoners resulted in ICERs of -$245, $268, and $477, respectively (Table 2 and Fig 3). Adopting a conservative maximum willingness-to-pay for healthcare interventions threshold of $28,000 per QALY gained, indicates that across prison settings and all LOS, DAA therapy is highly cost-effective, and cost saving as this level. The results are confirmed by the positive NMB at all levels of coverage, with an economic surplus of $6–10,000 per treated prisoner at 25% coverage, $19–20,000 per treated prisoner at 50% coverage, and over $40,000 per treated prisoner at 90% coverage (Table 2).

## Discussion

Using a dynamic model of HCV transmission, compared to treating 10% of HCV-infected prisoners, the incidence of HCV infection in NSW prison was reduced by 26% to 70% depending on the average LOS in prison and the uptake of DAA therapy. Taking into account the cost of treatment and lifetime care costs of living with HCV, our model indicates that DAA therapy is increasingly cost-effective with increasing coverage in prisons among all LOS and at least 25% coverage of HCV-infected inmates. All ICERs for an HCV-infected prisoner were below $500 when life-long health-related quality of life were considered which is lower than the Australia’s willingness-to-pay threshold $28,000 per QALY gained, and thus considered good value for money in terms of healthcare expenditure.

Most of the benefit from treatment in prisons is realized in the community following release. Our model therefore not only looked at the reduction in HCV prevalence in prison, but also the long-term cost, health-related quality of life and cost-effectiveness of DAA therapy when prisoners are released back to the community. The fact that much of the costs associated with treatment is carried by the state prison budgets (in our study Corrective Services NSW and Justice Health & Forensic Mental Health Network) rather than national healthcare budget suggests that prison budgets should be compensated for the health and custodial infrastructure costs of delivery of the DAA treatment, where drug costs are covered by the Commonwealth Government of Australia.

Ensuring access to treatment for large numbers of individuals with chronic HCV infection has put prisons firmly in the spotlight as a setting in which to identify and treat large numbers of hard-to-reach individuals impacted by HCV [23]. International guidelines recommend prioritization of the HCV treatment in PWID and prisoners, and that current injecting drug use should not be a contraindication [38]. Even in the constrained public healthcare system in the USA, the importance of HCV screening and treatment in prison can reduce the increasing incidence of new infections and cases of advance liver diseases and liver-related deaths [39] has been highlighted and considered “…a reasonable expenditure for correctional systems”, and that doing so “…may be a worthy target for public health dollars” [40].

To our knowledge, only one other study has specifically investigated the cost-effectiveness of DAA treatment in the prison setting [41]. This study used a Markov based model, and also concluded that DAA treatment was cost-effective at $25,000-$30,000 per QALY gained. However, this study compared traditional interferon-based therapies with 3-drug regimens that included the DAA (sofosbuvir) [41].

The strength of this study is that it investigates several treatment scenarios in prison and included the long-term cost and outcomes both in prison and the community associated with HCV treatment and care. A limitation of this study is that is does not track reinfection of those treated, that is, it assumes that once cured individuals it goes back to the susceptible population. The rates of reinfection in inmates are relatively high, estimated to be 5.27 cases per 100 person years under older interferon-based treatments [42]. However, given that acceptability DAA treatment and shorted treatment time needed to reach sustained virologic response (SVR), reinfections are likely to be lower than this [43]. The model did not consider future pharmaceutical developments during the thirty years prison time. It is possible that less costly treatments will become available during this time. Another limitation of our study is that we did not model the effect of treatment as prevention, and indeed prevention is essential to optimizing the benefits for HCV treatment to the population. A number of modeling studies have indicated that even moderate levels of treatment can lead to a significant reduction in the prevalence and incidence of HCV infections [44–47]. Therefore, our cost-effectiveness estimates are likely to be conservative, and thus even more cost-effective if the prevention of incident HCV cases in the population were included. Our model did not include testing strategies which can play an important role in detecting people who are living with HCV but are undiagnosed. A recent modeling study suggests that increased testing is important to eliminate HCV transmission in Australia [48]. Future work should also include modeling of screening strategies, linkage to HCV care, and the post-cure management to minimize reinfection of those who are cured, and the impact of HCV treatment in female versus male prisons.

In conclusion, treating HCV-infective prisoner is highly cost-effective and should be a priority for the global HCV elimination efforts.

## Supporting information

S1 File (DOCX)

## Abbreviations

HCV: hepatitis C virus
DAA: direct-acting antiviral
CI: confidence intervals
PWIDs: people who inject drugs
NSW: New South Wales
LOS: length of stay
QALYs: quality-adjusted life years
AUD: Australian dollars
AR-DRG: Australian Related fined Diagnosis Related Groups
ICER: incremental cost-effectiveness ratio
PSA: probabilistic sensitivity analysis
NMB: Net Monetary Benefit
SVR: sustained virologic response
