# Supplementary material for: Hepatitis C treatment strategies in prisons: A cost-effectiveness analysis
Source: PLoS One. 2021 Feb 11;16(2):e0245896. doi: 10.1371/journal.pone.0245896 (PMC7877645; doi:10.1371/journal.pone.0245896)
Supplement: S1 File — (DOCX) [file pone.0245896.s001.docx]

**Hepatitis C treatment strategies in prisons: a cost-effectiveness analysis**

**Supplementary material**

Austhors: Jisoo A. Kwon*^1^, Georgina M. Chambers*^2^, Fabio Luciani^3^, Lei Zhang^1,4^, Shamin Kinathil^1^, Dennis Kim^1^, Hla-Hla Thein^5^, Willings Botha^6^, Sandra Thompson, Andrew Lloyd^1^, Lorraine Ape, Richard T Gray^1^, Tony Butler^1^

^1^The Kirby Institute, UNSW Sydney, Sydney, New South Wales 2052, Australia

^2^National Perinatal Epidemiology and Statistics Unit (NPESU), Centre for Big Data Research in Health and School of Women’s and Children’s Health, UNSW Sydney, Sydney, New South Wales 2052, Australia

^3^School of Medical Sciences, Faculty of Medicine, UNSW, Sydney, NSW, Australia

^4^The Melbourne Sexual Health Centre, Alfred Health, 580 Swanston St, Carlton, Melbourne, VIC, 3053, Australia

^5^Toronto Health Economics and Technology Assessment Collaborative (THETA), Toronto General Hospital Research Institute, University of Toronto and University Health Network, Toronto, ON, Canada

^6^RTI Health Solutions, 3040 East Cornwallis Road, P.O. Box 12194, Research Triangle Park, NC, 27709, USA

***Joint first authors**

**Supplementary tables**

**S1 TABLE** Parameters describing HCV transmission and treatment

| **Type of parameter** | **Description** | **Average*** | **95% CI** | **Reference** |
| --- | --- | --- | --- | --- |
| HCV prevalence  in prison | HCV Ab+ve prevalence in PWID (%) | 73.7 |  | ^1^ |
|  | HCV Ab+ve prevalence in non-injectors (%) | 7.1 |  | ^1^ |
|  | HCV Ab+ve that are HCV RNA+ve in prison population (%) | 67 |  | Assumption |
| Infection rates /  Natural History HCV | Per event probability of HCV transmission | 0.0057 | [0.003–0.010] | ^2^ |
|  | HCV Clearance rate (year) | 0.25 | [0.21–0.30] | ^3^ |
|  | Acute -> F0 (TP) | 0.180948 | - | ^4^ |
|  | F0 -> F1 (TP) | 0.11604 | [0.059–0.228] | ^4^ |
|  | F1 -> F2 (TP) | 0.08496 | [0.065–0.110] | ^4^ |
|  | F2 -> F3 (TP) | 0.085 | [0.049–0.147] | ^4^ |
|  | F3 -> F4 (TP) | 0.131 | [0.053–0.319] | ^4^ |
|  | F4 -> Decompensated Cirrhosis (TP) | 0.031 | [0.020–0.096] | ^4^ |
|  | F4 -> Hepatocellular Carcinoma (TP) | 0.010 | [0.009–0.039] | ^4^ |
|  | Decompensated Cirrhosis -> Hepatocellular Carcinoma (TP) | 0.070 | [0.041–0.103] | ^4^ |
| Death rates | Death rate in prison | 0.108 | [0.042–0.175] | ^5^ |
|  | Decompensated Cirrhosis -> Liver Related Death (TP) | 0.039 | [0.033–0.046] | ^6^ |
|  | Hepatocellular Carcinoma -> Liver Related Death (TP) | 0.176 | [0.134–0.231] | ^6^ |
| Treatment parameters | SVR rate DAA (%) | 90-95 |  | ^7–10^ |
|  | SVR rate IFN (%) | 60 |  | ^11^ |
|  | IFN based treatment coverage (%) | 10 |  | Assumption |

*Rates are per year, otherwise stated

HCV, hepatitis C virus; AB, antibody; +ve, positive; PWID, people who inject drugs; RNA, ribonucleic acid; CI, confidence interval; TP, transition probability per year; SVR, sustained virologic response; DAA, direct-acting antiviral; IFN, Interferon

**S2 TABLE: Healthcare costs of treatment and care and health state utility values**

| **Annual costs of HCV care per person*** | **mean cost (AUD$)** | **LB** | **UB** |
| --- | --- | --- | --- |
| Precirrhosis stage of chronic hepatitis C (fibrosis stage 0 to 3) -all years | $550 |  |  |
| Compensated cirrhosis (fibrosis stage 4) | $850 | $824 | $875 |
| Decompensated cirrhosis (liver failure) - includes ascites, variceal haemorrhage and hepatic encephalopathy | $22,715 | $21,693 | $23,737 |
| Hepatocellular carcinoma | $23,660 | $19,402 | $27,919 |
| Delivery of Treatment in a prison setting | $666 | $633 | $700 |
| DAA interferon free therapy | $15,000 | $14,250 | $15,750 |
| **Health-state utilities and quality of life in hepatitis C patients**^12^ | Mean utility | Lower bound | Upper bound |
| Australian population norms (spontaneous viral clearance, never infected) | 0.93 | 0.928 | 0.932 |
| Sustained virological response | 0.92 | 0.9 | 0.94 |
| Acute HCV infection | 0.85 | 0.83 | 0.87 |
| Precirrhosis (Fibrosis stage 0 to fibrosis stage 3) | 0.85 | 0.83 | 0.87 |
| Compensated cirrhosis (Fibrosis stage 4) | 0.76 | 0.7 | 0.79 |
| Decompensated cirrhosis (liver failure) | 0.69 | 0.44 | 0.69 |
| Hepatocellular carcinoma (HCC) | 0.67 | 0.6 | 0.72 |
| Liver transplantation | 0.77 | 0.57 | 0.77 |

***** Unit costs were obtained from the Australian Medical Benefits Scheme (MBS) (assuming 100% government benefits) for medical services, procedures, and pathology tests. Medication costs were sourced from the Australian Pharmaceutical Benefits Scheme (PBS).^13,14^ Inpatient medical procedures and stays were sourced from the National Hospital Cost Data Collection using Australian Related fined Diagnosis Related Groups (AR-DRG 5.1).^15^

**References**

1. Butler T, Simpson M. *National Prison Entrants’ Blood-Borne Virus Survey Report 2004, 2007, 2010, 2013, and 2016*.; 2017.

2. Boelen L, Teutsch S, Wilson DP, et al. Per-event probability of hepatitis C infection during sharing of injecting equipment. *PLoS One*. 2014;9(7):e100749. doi:10.1371/journal.pone.0100749

3. Micallef JM, Kaldor JM, Dore GJ. Spontaneous viral clearance following acute hepatitis C infection: a systematic review of longitudinal studies. *J Viral Hepat*. 2006;13(1):34-41. doi:10.1111/j.1365-2893.2005.00651.x

4. Kwon JA, Anderson J, Kerr CC, et al. Estimating the cost-effectiveness of needle-syringe programs in Australia. *AIDS*. 2012;26(17):2201-2210. doi:10.1097/QAD.0b013e3283578b5d

5. Australian Institute of Criminology. *National Deaths in Custody Program: Deaths in Custody in Australia 2016–17*. Canberra, Australia; 2019.

6. Hallager S, Ladelund S, Christensen PB, et al. Liver-related morbidity and mortality in patients with chronic hepatitis C and cirrhosis with and without sustained virologic response. *Clin Epidemiol*. 2017;9:501-516. doi:10.2147/CLEP.S132072

7. Gane EJ, Stedman CA, Hyland RH, et al. Efficacy of nucleotide polymerase inhibitor sofosbuvir plus the NS5A inhibitor ledipasvir or the NS5B non-nucleoside inhibitor GS-9669 against HCV genotype 1 infection. *Gastroenterology*. 2014;146(3):736-743 e1. doi:10.1053/j.gastro.2013.11.007

8. Lawitz E, Poordad FF, Pang PS, et al. Sofosbuvir and ledipasvir fixed-dose combination with and without ribavirin in treatment-naive and previously treated patients with genotype 1 hepatitis C virus infection (LONESTAR): an open-label, randomised, phase 2 trial. *Lancet*. 2014;383(9916):515-523. doi:10.1016/S0140-6736(13)62121-2

9. Poordad F, Lawitz E, Kowdley K V, et al. Exploratory study of oral combination antiviral therapy for hepatitis C. *N Engl J Med*. 2013;368(1):45-53. doi:10.1056/NEJMoa1208809

10. Feld JJ, Jacobson IM, Hezode C, et al. Sofosbuvir and Velpatasvir for HCV Genotype 1, 2, 4, 5, and 6 Infection. *N Engl J Med*. 2015;373(27):2599-2607. doi:10.1056/NEJMoa1512610

11. Roberts SK, Weltman MD, Crawford DH, et al. Impact of high-dose peginterferon alfa-2A on virological response rates in patients with hepatitis C genotype 1: a randomized controlled trial. *Hepatology*. 2009;50(4):1045-1055. doi:10.1002/hep.23130

12. Hagan LM, Sulkowski MS, Schinazi RF. Cost analysis of sofosbuvir/ribavirin versus sofosbuvir/simeprevir for genotype 1 hepatitis C virus in interferon-ineligible/intolerant individuals. *Hepatology*. 2014;60(1):37-45. doi:10.1002/hep.27151

13. Australian Government: Department of Health. MBS Online: Medicare Benefits Schedule. http://www.mbsonline.gov.au/internet/mbsonline/publishing.nsf/Content/Home. Published 2020.

14. Australian Government: Department of Health. The Pharmaceutical Benefit Scheme. https://www.pbs.gov.au/browse/medicine-listing.

15. Commonwealth of Australia. *National Hospital Cost Data Collection Australian Public Hospitals Cost Report, Round 11*.; 2015.
